# Senior management’s understanding of and response to climate goals in healthcare: a qualitative study in a Swedish hospital

**DOI:** 10.1136/bmjopen-2025-112882

**Published:** 2026-04-28

**Authors:** Pamela Mazzocato, Henna Hasson, Sissel Kulstadvik, Mariel Taxén, Charlotta Sävblom, Linda Sturesson Stabel

**Affiliations:** 1 Department of Learning, Informatics, Management, and Ethics, Medical Management Centre, Karolinska Institutet, Stockholm, Sweden; 2 Södertälje Hospital, Södertälje, Region Stockholm, Sweden; 3 Center for Epidemiology and Community Medicine, Stockholm, Region Stockholm, Sweden

**Keywords:** Climate Change, Hospitals, Health policy, QUALITATIVE RESEARCH, HEALTH SERVICES ADMINISTRATION & MANAGEMENT

## Abstract

**Abstract:**

**Background:**

Healthcare is a major contributor to greenhouse gas emissions, and sector-specific policies and goals are recommended as governance tools. Despite leadership playing a critical role in reducing emissions, there is limited empirical research on how healthcare managers perceive and navigate climate goals and actions.

**Objective:**

To explore how members of a hospital’s senior management team understand and respond to climate goals.

**Design:**

Qualitative study design, semi-structured interviews were conducted, and data were analysed thematically.

**Setting:**

A hospital in Sweden.

**Participants:**

Members (n=15) of the hospital’s senior management team.

**Results:**

Five themes were identified. Senior managers recognised the importance of climate goals (theme 1), but their understanding of these varied—from perceiving them as concrete and actionable to abstract and irrelevant at the departmental level (theme 2). Climate goals were described as both visible and invisible (theme 3), and as both in alignment and in competition with other organisational goals (eg, patient safety, budget constraints) (theme 4). There was a common understanding that they, and the hospital, could do more to reduce emissions but knowledge gaps, limited resources, monitoring challenges, as well as systemic constraints, challenged advancement (theme 5). These dynamics led to two co-existing patterns of responses: a *virtuous cycle* of climate action and a *vicious cycle* of climate inaction in which uncertainty prevailed.

**Conclusions:**

Climate goals can act as both catalysts and inhibitors to climate actions in hospitals, depending on how they are understood and operationalised by senior managers. Policymakers and healthcare system leaders must address the uncertainty surrounding climate goals to advance climate action.

**STRENGTHS AND LIMITATIONS OF THIS STUDY**

- A vast majority of members from a hospital senior management team were included.
- In-depth interviews allowed rich data on how healthcare leaders understand and respond to climate goals.
- Results are based on the members of one hospital senior management team only.

## Introduction

 The healthcare sector, designed to promote health, paradoxically poses a threat to public health through extensive greenhouse gas (GHG) emissions that drive climate change.^1^ The healthcare sector is responsible for 4.2–5.2% of the global GHG emission,^2 3^ and hospitals are major contributors.^4 5^ To mitigate healthcare negative impact on climate, sector-specific goals are often recommended^6^ and used as a governance tool to reduce emissions. For example, Providence Health and Services, which operates 52 hospitals across the USA, aims to become carbon negative,^7^ the National Health Service England aims for net zero,^8^ and so does the network Healthcare Without Harm that gathers thousands of hospitals.^9^ In Sweden, regional agreements, in alignment with the national mission to achieve net-zero emissions by 2045,^10^ mandate that hospitals should actively work to reduce their environmental impact by setting hospital-specific climate goals. While the use of healthcare sector-specific goals has been recommended as a strategy to reduce GHG emissions,^6^ healthcare falls behind other service sectors to reduce emissions.^11^ Calls to actions underscore that more can and must be done.^12^

Healthcare leaders at all levels, for example, governing boards, executives, and clinical leaders are expected to contribute by translating sector-specific goals into organisational goals to reduce GHG emissions, put strategic climate actions into place, monitor progress and designate responsible parties to create organisational policies.^13 14^ Leaders are expected to lead sustainability initiatives within healthcare facilities as a strategic necessity, addressing internal conflicts while promoting environmental responsibility.^15^ Moreover, leaders can increase organisational capacity by ensuring resources and competencies,^16^ fostering a culture of sustainability^13^ and raising awareness among staff.^17^

Despite the large number of expectations posed on healthcare leaders to reduce GHG emissions^8 13 14 16 18^ and the extensive literature on pathways for emission reduction linked to climate goals,^16 19^ few empirical studies explore healthcare leaders’ efforts to reduce emissions^20^ and seldom depart from the perspective of leaders with formal managerial responsibilities. Empirical research has foremost focused on staff perspective and the barriers they may face.^21^,^23^ Given the strong mandate and decision-making power that managers, particularly senior management team members, hold in healthcare, it is important to explore managerial perspectives.

Research conducted in other sectors shows that managers’ sensemaking, beliefs and values influence corporate environmental strategies and actions.^24 25^ Tensions have been found between economic, social and environmental goals.^26 27^ Managers’ may choose not to act due to uncertainty in trade-off situations.^26 27^ Managers’ understanding guides, sometimes unconsciously, their behaviour and decisionmaking by reinforcing habitual thinking.^28 29^ Without examining and adjusting them, organisations may struggle to improve.^30^ The complex interplay of multiple organisational goals and the challenges of addressing them simultaneously may influence senior managers’ decision making and ability to drive positive change.

Although healthcare managers are recognised as playing a pivotal role in reducing healthcare emissions, there is limited, if any, empirical knowledge about their understanding of climate goals. Given the existing practice of setting sector-specific and organisation-specific climate goals, the potential influence of senior managers in enabling the achievement of these targets, and the likelihood that their understanding shapes decision-making and organisational climate actions, this study explores how hospital senior managers understand and respond to climate goals.

## Methods

This qualitative study is based on interviews with members of a senior management team in a Swedish hospital, hereafter referred to as senior managers.

### Setting

The Swedish healthcare system is decentralised with a regulating national legislation for policy and 21 regional independent bodies that are responsible for financing (ie, taxes from the citizens), organising and providing healthcare.^31^ Besides healthcare, the regions are responsible for regional public transportation, development and cultural activities.

In the region where this study was conducted, a purchaser-provider model is applied and the purchaser, that is, the Regional Health and Medical Administration, contracts the provider for volume of healthcare activities to be delivered and sets specific goals that the providers should achieve for full payment. In the same region, the national climate policy outlined by the Swedish Parliament^10^ was translated and defined as regional long-term goals and specified into regional climate and sustainability goals by the Regional Council. These goals were further translated into yearly pay-for-performance indicators set by the Regional Health and Medical Administration. These pay-for-performance goals were then turned into hospital-specific goals approved by the hospitals’ senior management team members (table 1). If the goals were not achieved by the end of the year, then the hospital would not be reimbursed for up to 0,5% of the total contract value.

**Table 1:** Overview of regional and hospital climate goals

Levels of goal setting	Climate-related goals						
Regional long-term goals set by the Regional Council.	Annual direct greenhouse gas emissions will be less than 1.5 tons per capita, and consumption-based greenhouse gas emissions will be reduced by 50%. Annual energy use per capita will decrease continuously to below 16 MWh, and the region’s energy production will be 100% renewable. The share of public transport in motorised travel will increase by 5 percentage points compared with 2015, with at least 70% of all trips within the county being made by walking, cycling and public transport. The share of cycling will reach 20% in accordance with the regional cycling plan. Household waste will be reduced to a maximum of 360 kg per person per year, and at least 70% of this waste, including food waste, will be recycled.						
Regional climate and sustainability goals that apply to all sectors governed by the Regional Council.	All operations in the region have by 2035 minimised their emissions of harmful substances.	The total climate impact of the Region should be reduced by 50% by 2030 compared with 2019.	Circular flows should be established by 2035.	Waste from patient meals should decrease by 5%.			
Pay-for-permanence hospital goals set by the Regional Health and Medical Administration.		The caregiver should conduct an active work to reduce the impact on the environment and the climate from the use of materials and products.	The caregiver should conduct an active work to reduce the impact on the environment and the climate and strengthen circular flows.	The caregiver should work actively to reduce and maintain a low waste of food.	The caregiver should work to contribute to an increase in biological diversity through the purchase of organic foods for patient meals. Organic food products should meet the criteria on organic production of agricultural products according to regulation.	The caregiver should work actively for an efficient and patient-safe drug management to reduce the impact of pharmaceuticals on the environment.	The caregiver should have a certified environmental management system according to ISO 14001 standard, EU Eco-Management and Audit Scheme (EMAS) or equivalent.
Local hospital environmental goals set by the hospital management team.		Reduce the use of materials and products to reduce the consumption-based greenhouse gas emissions.	Strengthen circular material flows to reduce the consumption-based greenhouse gas emissions.	Food waste from patient meals should have decreased by at least 45% compared with 2017.	The proportion of organic food in the served patient meals should be at least 50% (in monetary value) or 35% (by weight).	Reduce disposal of pharmaceuticals.	The hospital should conduct audits with planned intervals according to the environmental management system (ISO 14001).

The goals investigated in this study were the ones labelled ‘local hospital climate and environmental goals’ in table 1.

The hospital was selected for three main reasons: it represented a typical average size Swedish hospital; it operated under standard regional agreements to achieve set climate goals; and the researchers were given full access to the hospital. A description of the hospital and how climate goals were managed is presented in table 2.

**Table 2:** Overview of the organisational context to climate and environmental goals

The hospital	Senior management team	Dedicated functions	All employees
Approx. 1300 full-time employees. 200 beds. Operates as a combined emergency and community hospital and offers a wide range of healthcare services, both acute and planned. Certified according to ISO 14001 and operated under an environmental management system (EMS).	Yearly, the senior managers developed an action plan to achieve its strategic goals in line with regional goals, including climate goals. Senior managers were responsible for overseeing EMS-related processes. Environmental reviews and progress towards the regional goals were reported in a senior management meeting twice a year by the hospital’s sustainability strategist.	A part-time sustainability strategist supported the senior management team in defining actions to meet these goals. At the unit level, appr. 50 clinical staff members had an assignment as environmental agents to support practical actions to achieve the goals.	All employees were expected to attend a 60-minute online course on sustainability, with specific components for managers.

### Participants

Participants were purposively selected to represent the senior management team members of the hospital. The team consisted of 19 senior managers including a hospital director, two chief medical officers, a chief nurse officer, directors of clinical departments and directors of support functions (eg, HR, finance, communication, IT). The participants had the formal responsibility to define the hospital yearly climate goals and actions. The senior managers who were head of clinical departments or support functions were responsible for defining the specific activities to conduct within their department or unit.

The study was first presented at a senior management team meeting by the first and last authors, and an email was thereafter sent individually to each member for recruitment. Of the 19 members, 15 accepted to participate in the study.

### Data collection

The first and last author developed a semi-structured interview guide (see online supplemental material), which was tested with two people outside the study, after which minor modifications were made, that is, the number of questions was reduced. The guide was organised into sections covering the interviewees’ professional background, perceptions and experiences of the climate goals and actions, as well as facilitators and barriers. The interviews were conducted in Swedish and individually by one or two of the authors. 13 interviews were conducted at the hospital, and two online via Teams. Interviews lasted between 36 and 83 min (mean, 52 mins), were audio recorded, and transcribed. Written consent for participation was obtained. The interviews were performed in October 2023–February 2024.

### Data analysis

Data were analysed thematically.^32^ Transcripts were read to familiarise with the data, and ideas about the content were noted, discussed and coded. Each interview was coded by at least two of the authors, Excel spreadsheets were used for this phase. To sort the data, codes were moved to the collaboration platform Miro. Codes were discussed and grouped into categories and themes by several authors collaboratively. Multiple meetings were held in which the codes, categories and themes were discussed iteratively by the authors. The first and the last author reviewed the final themes and developed a model that synthesised the findings.

During the analysis process, the researchers engaged in reflexivity^33^ to unfold their perspectives, pre-understandings and assumptions influencing their interpretations. This was particularly valuable as two of the authors were also members of a senior management team. The results of the qualitative analysis were critically discussed by all authors. The results were presented at a senior management team meeting to allow for additional reflections and insights.^34^ Quotations were selected and translated from Swedish to English.

## Results

The 15 senior managers had been part of the management team from a few months to 7 years (mean 2.8 years), and 13 were female and two were male. The analysis of the interview data resulted in five themes. The first reflected a shared understanding that climate goals were important. Three themes revealed divergent understandings: climate goals were perceived as both clear and abstract (theme 2), visible and invisible (theme 3), and aligned with, yet sometimes in conflict with, other organisational goals (theme 4). The fifth theme concerned the perception that they were doing what they could but perceived constraints that hindered climate actions (theme 5). The results include first a description of each theme, followed by a model that synthesises the findings.

### Climate goals are important, and the hospital can make a difference

Senior managers considered the hospital climate goals important as they knew that the hospital had emissions and thereby had an impact on climate. They identified various sources of emissions, such as travel, food waste, production and washing of clothing, material supply, transport of products and medicine, energy consumption for technical equipment, nitrous oxide and other anaesthetic gases, and single-use plastic products. When the sources could be addressed, senior managers described that the hospital could make a difference. They viewed the hospital as an important actor for mitigating climate change based on its large emissions and the hospital’s role in society.

It is clear that we have a substantial impact. Moreover, we are a public administration, and symbolically, it has a significant impact that we are also trying to take responsibility for the whole and for society. (participant 11)

Actively engaging in climate actions was viewed as a potential asset for employer branding, helping to attract new staff. Showing that part of the job was value-driven could send positive signals and instil a sense of pride among employees.

Senior managers expressed that climate goals were helpful to shed light on the climate crisis, but the financial compensation tied to their achievement was perceived as a ‘stick’ rather than a ‘carrot’, making it challenging to use as a governance tool. If the goals were not achieved, the hospital would lose up to 0,5% of the total contract value, which could refrain senior managers from setting overly ambitious goals, out of concern for potentially missing out on goal-related compensation.

Yes, budget. It is a very strained situation, so I think people will be very cautious about setting goals that become very, very difficult to achieve […]. It is not really the time for that now. (participant 2)

### From clear to abstract

Among the senior managers, the perceptions of the goals varied from being clear to abstract. When perceived as clear, self-evident and relevant to the department, goals were experienced as rather unproblematic to address and to be included in action plans at the department level. In contrast, some goals were also perceived to be at a high level of abstraction, too vague, unclear or too narrow and thereby irrelevant to specific departments. When the goals were perceived as abstract, too narrow or irrelevant, it contributed to managers’ uncertainty on how to address them. Senior managers found these challenging to work with, that is, to break them down, translate them into departmental actions, and to quantify.

When you hear them [the climate goals], you think ‘Oh, how does this concern us?’ It feels a bit like they might be at a very high level. But of course, we probably need to bring it down somehow. So those goals might, when you are sitting in the hospital’s management team, feel like, ‘Who is going to handle this?’ It is a bit distant. (participant 5)

When senior managers perceived the goals as irrelevant, it was typically in relation to their own department rather than to the hospital, which made it challenging to identify appropriate actions. Abstract goals were seen as distant from daily operations and beyond the influence of staff, reducing motivation and engagement.

### From visible to invisible

There were varied opinions on the prominence of climate goals at senior management team meetings and in the hospital overall. Climate goals and issues were perceived as visible and ever-present by some while others perceived goals as invisible or peripheral. Senior managers often felt that their efforts to achieve the goals were not sufficiently visible to staff.

Visibility and presence of climate goals increased in some situations, often related to specific organisational roles. The sustainability strategist was perceived as a reminder of climate goals and actions, for instance, at senior management team meetings when reporting on the environmental review and the status of climate goals. At department and unit levels, environment was a mandatory recurring topic in monthly workplace meetings due to the EMS. Furthermore, during clinical work, climate issues could become visible for managers.

I think about climate issues when working clinically, especially the enormous material consumption involved in working closely with patients. (participant 5)

Except for situations with physical reminders, climate goals were in general mentioned as easy to forget even when they should be present. A concern was raised that the absence of a dedicated climate representative in the senior management team, such as a sustainability strategist, would lead to climate issues being forgotten or marginalised. At unit or departmental level, the environment item in the monthly workplace meetings was often mentioned to be sidelined in favour of other matters that were deemed more pressing. Sometimes the item was left empty because managers were uncertain on what to discuss and how to discuss it. Without regular reminders, climate goals and actions became overshadowed by other priorities perceived as more directly linked to the hospital’s core mission and risked losing momentum.

### From alignment to conflict with other organisational goals

Overall, climate goals were seen as aligned with the broader organisational purpose. However, there were instances where pursuing climate goals was perceived to compete with other organisational goals. Managers tended to view each goal separately and often took a short-term perspective as organisational goals were set annually.

An example when climate goals aligned with other goals was the establishment of a storage for the rare-supply-of-medicines which reduced unnecessary medication purchases and thereby reduced costs. Similarly, focusing on proper use of plastic gloves was considered beneficial for climate but also for hygiene and finance.

In contrast, some situations were described when actions that would benefit climate goals could compromise staff’s work environment. For instance, optimising facility use through shared working stations was a concern. Another example of conflicting goals was the reduction of single-use-products, which could be beneficial for the climate but was perceived to be potentially harmful for patient safety.

Disposable surgical clothing, for instance, is discarded after a work shift. It cannot be environmentally correct. While it is likely excellent for patient hygiene, it cannot be beneficial overall. (participant 9)

Senior managers emphasised a cautious approach when making decisions related to climate goals, preferring to be ‘better safe than sorry’, particularly when actions could impact staff work environment or patient well-being and safety. For example, there was uncertainty to replace patient meals with vegetarian options out of concern that they might not be well received by the patients.

When organisational goals were considered in relation to one another, climate goals often competed with other priorities, leading to trade-offs where the climate perspective frequently took a backseat. Senior managers described how urgent concerns, such as high production volumes and staff shortages, often overshadowed climate goals, resulting in these goals being sidelined and given lower priority. Despite these challenges, there were instances where climate priorities were upheld even at a higher cost, for instance, when an expensive compressor was bought to enable the circular flow of plastic aprons, an action which senior managers had pushed for.

### From doing what we can to focus on constraints

Senior managers mentioned many ongoing climate actions but mentioned that they, or the hospital as a whole, could do more to reduce their negative impact on climate. Senior managers had ideas for potential climate actions but also expressed challenges about how to advance beyond the efforts going on. Challenges were linked to several constraints, such as knowledge to identify the ‘right’ actions, resources and systems for monitoring and follow-up, and the constraints of operating within a larger healthcare system.

When choosing climate actions, senior managers reflected on the value of being pragmatic and the need to sometimes rely on common sense rather than overthinking, but foremost they wanted to be sure to choose the ‘right’ and most effective climate actions. They were however uncertain about the footprint of different products, the impact of different actions and what actions to focus on. Daily behavioural changes were mentioned to be required to make progress as collective efforts could add up over time, yet there were doubts about the significance of such efforts.

Well, maybe the impact is not that big, or—I do not know—but at the same time, it is something that happens every day. Even if the volumes are not that large each day, it obviously adds up over the course of a whole year. (participant 1)

Senior managers often expressed that they lacked knowledge or competence related to climate issues and expressed having difficulties in accessing relevant information.

Resources were also perceived to be a constraint. Senior managers reported limited access to economic resources, staff and time to allocate personnel for climate actions and to monitor their effects, particularly since data had to be retrieved manually. They also noted the absence of adequate systems and tools to track the impact of climate actions, and identifying relevant metrics was often challenging.

I also do not really know what resources I have to achieve the climate goals, other than the money I am expected to spend in a sensible way, I suppose. (participant 9)

Senior managers often emphasised the importance of ensuring that investments in climate actions should have a meaningful impact, without causing rebound effects or unintended consequences that could negatively affect patient care. Despite uncertainty about the effects of climate actions, managers described taking actions even when outcomes were unclear. For example, one senior manager explained that the department worked to reduce nitrous oxide, although the impact was vague, since you cannot say, “because we did this, the ozone hole became ten centimetres narrower” (participant 3).

Senior managers often mentioned the constraints of operating within a larger healthcare system and some endeavoured to do what they could within their circle of influence. When climate goals and actions required joint efforts from multiple actors, such as suppliers, climate actions were perceived as more difficult to implement. They mentioned being locked in procurements and being uncertain about what was going on at the regional level.

We are kind of part of a bigger context, especially when it comes to issues like these. And we cannot really start working on certain matters just here at the hospital when it comes to sustainability. It has to be coordinated with the larger system. So, we feel that we have to wait a bit for that. For now, we are doing what we have to do. (participant 2)

The perceived constraints had several consequences for climate actions. Informed decision-making was hampered and managers sometimes felt that it was difficult to contribute, and mentioned themselves to “fumble, and choose actions not being the sharpest” (participant 5). As a result, efforts often focused on the so-called ‘low-hanging fruits’, and goals were set so that the managers would be confident they could achieve them. Unlike other organisational objectives, climate goals were not monitored regularly, which led to a loss of momentum and, in some cases, left climate initiatives on hold.

### Responses to climate goals: the virtuous cycle of climate action and the vicious cycle of climate inaction

**Figure 1:**
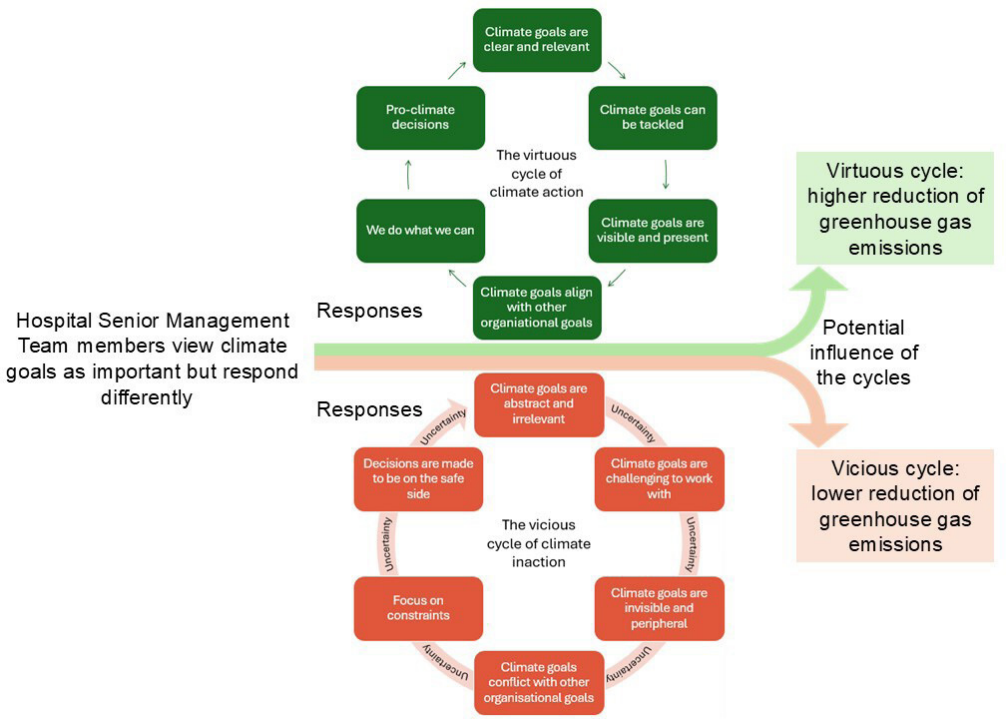
Hospital senior management team members’ divergent understanding of, and responses to, climate goals: The virtuous and vicious cycle of climate (in)action, and how this potentially influences emission reduction.

While all senior managers expressed a belief in the importance of climate goals, their perceptions of how to act on these goals, and whether they were actionable, varied. These differing perceptions can be synthesised into two contrasting cycles of responses: a virtuous cycle of climate action and a vicious cycle of climate inaction (figure 1). The cycles, which can co-exist within the same organisation, should not be viewed as a chain of reaction in which each box necessarily leads to the following box, but rather as patterns that may emerge starting from anywhere in the cycle. The two cycles of responses exemplify conditions that may enable or hinder senior managers’ ability to reduce GHG emissions, although this study did not evaluate the actual effects of these cycles on GHG emissions.

In the virtuous cycle, climate goals are regarded as important (theme 1) but also seen as concrete and relevant for the department level (theme 2). Senior managers can relate the goals to their departments’ operations and incorporate them into their action plans. Climate goals and actions are made visible (theme 3), for instance through the work of dedicated individuals who function as reminders. These reminders keep climate concerns top-of-mind and climate goals are seen as aligning with other organisational goals (theme 4), such as improving efficiency, reducing costs and strengthening the hospital’s image as a responsible employer. This sense of alignment can foster climate engagement and justify resource investment. Even when the impact of specific climate actions is uncertain, managers feel empowered to do what they can and take (theme 5) pro-climate decisions.

In contrast, the vicious cycle begins with the same belief in the importance of climate goals (theme 1), but this belief is undermined by persistent uncertainty and constraining organisational conditions. Senior managers understand the goals as too broad, vague or narrowly defined to be actionable at the department level (theme 2). This disconnect makes it difficult for managers to identify appropriate measures or translate goals into concrete actions. Without visible reminders of climate goals, climate concerns get overshadowed by more immediate organisational priorities (theme 3). When climate goals are perceived to conflict with concerns related to patient safety, staff well-being or budgetary constraints, they are often deprioritised (theme 4). The lack of knowledge, data or systems needed to make informed decisions further constrain climate actions, especially when climate actions require collaboration with actors outside the hospital (theme 5). As a result, managers tend to set easily achievable goals or even climate actions risk being on hold.

## Discussion

This study contributes empirical insights into how hospital senior managers understand and respond to climate goals. Although climate goals are commonly recommended as governance tools to drive climate actions in healthcare,^6^ our findings demonstrate the divergent understanding and responses to climate goals, and the complexity and uncertainty associated with their practical use.

Our study suggests that senior managers’ understanding of the clarity and relevance of climate goals can trigger different responses, in this study outlined as two cycles, a virtuous and vicious, illustrating how climate goals may function as catalysts and inhibitors of climate actions. For example, when goals are perceived as abstract or lacking relevance to the department level and clinical operations, managers may deprioritise them even when they recognise their importance. In this way, climate policies and goals risk becoming symbolic gestures rather than strategic governance tools for transition. Policy makers and health system leaders must ensure goals that are actionable and relevant at multiple levels. The fact that climate goals could be understood as irrelevant for the specific department may be indicative of silo-thinking, that is, the senior managers primarily considered their own departments rather than taking a hospital-wide perspective. As members of the senior management team, managers are tasked with setting strategic priorities for the whole organisation. Internal fragmentation and siloed operations may inhibit the ability to take a hospital or societal perspective on climate actions,^15^ thus triggering the vicious cycle. To overcome silos and inaction, senior managers need to lead climate actions with a focus on multiple levels, namely their departments, the hospital and society. Fragmentation and silo-thinking suggest policy should promote cross-sectoral collaboration, for example, through sustainable procurement standards or the establishment of forums for collaboration and knowledge-sharing, both within the hospital and with external stakeholders, such as suppliers or regional transport companies.

A key insight from this study is the central role of uncertainty which reinforced the vicious cycle in shaping managerial decision-making for climate actions. We found uncertainty regarding how to translate goals into effective actions, how to evaluate impact and choose actions, how to prioritise climate goals amid competing organisational priorities and how to reconcile short-term goals with long-term environmental responsibility. These sources of uncertainty can be related to dimensions of uncertainty identified in the broader climate adaptation literature.^35^ Our results show how this uncertainty may be manifested in healthcare. Choice uncertainty, that is, uncertainty about which actions to take,^35^ was evident as managers felt they lacked knowledge into which climate actions had the greatest impact, which has previously been reported among healthcare professionals.^36^ While insufficient knowledge represents a barrier to implementing climate actions in healthcare,^37^ the literature offers a wide range of roadmaps and frameworks,^16^ potential solutions^617 38^,^40^ and best practices which are shared in networks.^2^ Ensuring that senior managers are aware of existing evidence-based strategies is a fundamental step in reducing hospitals’ negative impact on climate. Outcomes uncertainty, that is, uncertainty about the effects of actions,^35^ was attributed to the absence of tangible indicators and monitoring systems linking climate actions to reduced emissions and to the perceived alignment or misalignment between climate goals and other organisational priorities. Moreover, senior managers in some cases struggled to reconcile long-term climate goals with the short-term pressures of budget cycles, a form of temporal uncertainty, that is, uncertainty about when results will appear or when actions should occur.^35^ Similar tensions between organisational goals have been documented in healthcare.^41 42^ This study adds to the current literature by showing the tensions that managers may perceive when adding climate goals to more established organisational goals.

While we found examples of senior managers acting despite outcomes uncertainty as they relied on ‘common sense’, the multiple sources of uncertainty identified in this study could lead to risk-averse decisions, such as focusing on ‘low-hanging fruits’, which manifested predominately in the vicious cycle. While addressing low-hanging fruits has been suggested to be an opportunity for hospitals quickly wanting to implement and advance green goals,^13^ such actions may indicate that an organisation is only in a first environmental wave, with a potential to advance.^43^ Moreover, outcomes uncertainty^35^ can increase the perception that climate change is an abstract phenomenon (cf. ^44^) which in turn may increase the psychological distance that contributes to diminishing perceived relevance of climate goals and actions.^44 45^ While the consequences of uncertainty on managerial decision-making for climate actions remain unclear,^35^ becoming aware of the senior managers’ uncertainty may be important to avoid climate in-action.

Our results highlight the need for an overarching system-level approach to develop a coherent monitoring system. Despite the need for monitoring systems that link emissions to climate actions to inform decision-making and track progress over time (cf.,^46^,^49^) such systems are seldom implemented, creating barriers for environmental sustainability activities in healthcare organisations.^50^ Our results highlight the need for monitoring data being accessible and meaningful for organisational decision-makers. Monitoring systems could be integrated into organisational business intelligence platforms to provide data to decision-makers such as senior managers. A pragmatic approach could be to start by including emission data on sources where such data is already available and is related to hotspots (cf.,^47^) and to widely used products and resources, such as disposable gloves, medical gases, and energy consumption. However, compiling such information often requires a complex process of collecting data from multiple sources and IT systems, resulting in substantial administrative work in the absence of automated solutions. Consequently, allocating resources in the development of monitoring systems, beginning with measuring GHG emissions, is needed. As these systems become established, emission data could stepwise be linked to costs and potentially to health outcomes, for example, by applying approaches such as the mortality cost of carbon.^51^ Despite the inherent uncertainties in current emission measurement methods and estimation approaches, some degree of imprecision may need to be accepted to provide feedback to support those working to reduce emissions. Such monitoring systems could help senior managers who struggle to reconcile long-term climate goals with the short-term pressures of budget cycles. The integration of climate and sustainability aspects into governance systems could further help senior managers adopt a both–and logic^52^ that can make managers begin to see climate responsibility not as competing with but as enhancing other goals, such as population health.^53^ This integrative mindset could be further supported by adopting public value frameworks in hospital governance.^54^

The main limitation of this study is that participants were recruited from a single hospital, which restricts the transferability of the findings. While including participants from multiple settings could have enabled the exploration of a broader range of perspectives, focusing on one organisation allowed us to develop an in-depth and multi-faceted understanding of a complex issue in its real-life context, namely, how members of a senior management team understand and respond to climate goals.

Importantly, the findings illustrate that multiple understandings and experiences can coexist within the same organisation, that is, one organisation does not entail one shared understanding. These multiple perspectives are synthesised into a conceptual model—the virtuous and vicious cycles—which may be used to guide the design and analysis of future multi-case studies.^55^ Furthermore, the methods section provides a thick description of the study setting to support readers in assessing the transferability of the findings. The hospital is located within a regional healthcare system in which national climate policies are translated into regional climate and sustainability goals, which are in turn operationalised through annual pay-for-performance indicators for healthcare providers in the region, including the studied hospital. Studying this setting offers a particular advantage: it provides insights into a hospital system where climate goals are explicitly linked to financial incentives and reimbursement mechanisms, thereby illuminating both the opportunities and potential pitfalls associated with implementation. Given the novelty to focus on specifically hospital senior managers, a strength of the study is the contribution of insights on how healthcare’s managers understand and respond to climate goals, which is knowledge that can be used to strengthen the ability to reduce healthcare emissions. Two interviews were conducted digitally, which may have influenced the depth of these interviews, although we could not observe such effects. Eight interviews were conducted by two researchers, which could create a power imbalance. To decrease this risk, one researcher was the main responsible for conducting the interview. A strength of being two researchers was, however, also identified: the possibility for the other to complement with questions.

Future research could explore the use of the virtuous and vicious cycles as a practical tool to support climate actions in healthcare organisations. The cycles may be used to enhance managers’ understanding and foster collective sensemaking. Research could also seek to establish a link between managers’ role and effective climate actions. Further, studies across organisational settings are encouraged to enhance the transferability of the findings.

## Conclusion

Climate goals can help policymakers and clinicians to set focus on climate matters on the agenda for hospital senior managers. However, translating climate goals into effective climate actions remains fraught with challenges for top managers. The reduction of hospitals’ GHG emissions requires not only setting goals but actively managing how they are understood, operationalised, and monitored. Addressing the uncertainty that surrounds climate goals and actions should be a priority for policymakers and health system leaders to avoid the downward spiral of in-action.

## Supplementary material

10.1136/bmjopen-2025-112882 online supplemental file 1

## Data Availability

No data are available.
