# Supplementary material for: Senior management’s understanding of and response to climate goals in healthcare: a qualitative study in a Swedish hospital
Source: BMJ Open. 2026 Apr 28;16(4):e112882. doi: 10.1136/bmjopen-2025-112882 (PMC13140950; doi:10.1136/bmjopen-2025-112882)
Supplement: online supplemental file 1 [file bmjopen-16-4-s001.docx]

**Interview guide**

The interview guide covered the following topics and the questions served as starting points, with follow-up questions adjusted to answers.

Professional role and responsibilities

- Could you please describe ...
- your role within the hospital?
- how long you have been part of the hospital senior management team?
- the team’s overall mandate regarding organizational development and strategic priorities?

Perceptions of climate goals

- What are your thoughts on the healthcare sector’s climate impact?
- How do you perceive the climate goals that your organization is expected to meet?
- How would you describe the relevance or importance of these goals in relation to other strategic goals?
- In your view, what gives these goals legitimacy or priority, or makes them less prioritized?

Senior management team’s role to achieve climate goals

- How do you see the senior management team’s role in supporting or driving progress toward climate goals?
- What kinds of discussions about climate goals occur within the leadership group?
- Are there competing priorities that affect the team’s ability to address climate-related issues?
- What makes it easier or more challenging for the leadership group to follow through on climate ambitions?

Conditions for implementing climate actions

- From your perspective, what influence whether climate measures can be implemented successfully?
- Are there particular barriers that make it difficult to translate climate goals into practice?
- What factors or structures (formal or informal) help facilitate progress?
- Are some goals or activities more feasible or relevant than others?

Impact and effectiveness

- Which types of actions or interventions have the greatest potential to reduce the hospital’s emissions?
- Which measures implemented, which are most effective, and why?
- Are there actions that you think could be effective but are not currently pursued? What stands in the way?

Engagement and involvement

- How would you describe staff engagement in climate-related issues?
- Who tends to be involved in climate-related work and who is not, but perhaps should be?
- What factors make employee involvement easy or difficult?
- Are there existing forums, networks, or informal spaces that support engagement?

Support Needs and Organizational Improvements

- What kind of support would you as a leader need to work more effectively toward climate goals?
- Are there structures, tools, or forms of guidance that would make climate-related work easier?
- Looking broadly, what changes would strengthen the hospital’s capacity to meet climate goals?
