# Supplementary material for: Senior management’s understanding of and response to climate goals in healthcare: a qualitative study in a Swedish hospital
Source: BMJ Open. 2026 Apr 28;16(4):e112882. doi: 10.1136/bmjopen-2025-112882 (PMC13140950; doi:10.1136/bmjopen-2025-112882)
Supplement: Supplementary file 2 [file bmjopen-2025-112882.reviewer_comments.pdf]

PEER REVIEW HISTORY

BMJ Open publishes all reviews undertaken for accepted manuscripts. Reviewers are asked to complete a checklist review form and are provided with free text boxes to elaborate on their assessment. These free text comments are reproduced below.

ARTICLE DETAILS

Title (Provisional)

Senior management's understanding of and response to climate goals in healthcare: a qualitative study in a Swedish hospital

Authors

Mazzocato, Pamela; Hasson, Henna; Kulstadvik, Sissel; Taxén, Mariel; Sävblom, Charlotta; Sturesson Stabel, Linda

---

VERSION 1 - REVIEW

**Reviewer**	**1**
**Name**	**Runkle, Jennifer**
**Affiliation**	**North Carolina State University, North Carolina Institute for Climate Studies**
**Date**	**08-Dec-2025**
**COI**	**None**

Hospital top management's understanding of and response to climate goals in healthcare: a qualitative study

Manuscript ID: bmjopen-2025-112882

General Comments:
The thematic analysis and resulting themes effectively capture the practical challenges associated with setting and operationalizing climate goals within hospital systems, particularly those linked to financial incentives and reimbursement. The qualitative interviews provide a compelling picture of the real-world tensions between pursuing ambitious versus "low-hanging fruit" climate goals, the potential for unintended consequences, and the complexity of coordinating sustainability efforts across systems beyond the hospital itself. The manuscript also appropriately highlights the friction between climate action and competing organizational pressures such as economic constraints, staffing shortages, and immediate clinical priorities.
Although the paper only focuses on one hospital system, the key advantage is that goals have been adequately set and tied to reimbursement, much further than most hospital systems and results reveal a sobering picture of the pitfalls of implementation. This paper

will be of interests to BMJ's readership and help guide other health systems in the process of agreeing and acting upon climate goals.

Abstract:
The abstract is clear, well aligned with the study objectives, and accurately reflects the study design, key findings, and implications.

Introduction:
Fantastic introduction. The introduction is strong, well written, and comprehensive. It effectively situates the study within the global movement toward net-zero healthcare systems, clearly
articulates the leadership role of healthcare managers in reducing greenhouse gas emissions, and convincingly establishes the literature gap regarding decision-makers' perceptions and
implementation challenges. The rationale for the study is well justified.

Methods:
This paper cites an important case example of a Swedish Healthcare System where the national climate policy outlined by the Swedish Parliament, was translated and defined as regional long-term goals and specified into regional climate and sustainability goals by the Regional Council and further translated into yearly pay-for-performance indicators set by the Regional Health and Medical Administration. These pay-for-performance goals were then turned into hospital- specific goals and approved by the hospital; if goals were not achieved by the end of the year then reimbursement would be half of the total contract value. Top managers at the selected hospital system were responsible for environmental progress reviews and were selected to be in the study is well justified. The inclusion of reflexivity was an added advantage towards the study design, enhancing transparency.

Results:
Five themes emerged and are clearly described.

Figure 1: The figure is currently too small to read and would benefit from improved resolution and layout. In addition, the caption should be revised to better support the figure as a stand-alone element. Clarification of the intended audience (e.g., hospital leadership vs. the broader healthcare system) would also strengthen the figure's interpretability and impact

Discussion:
The discussion is thoughtful and appropriately emphasizes the need to move beyond siloed

thinking and uncertainty by implementing monitoring systems that link climate actions to emissions, population health, and costs. However, this recommendation reads as quite ambitious at present. The manuscript would be further strengthened by providing more concrete examples of what the input data for such monitoring systems might look like in practice and how these data could realistically be scaled to the hospital level. Additional consideration of feasibility, data infrastructure, and resource requirements would enhance the policy relevance of this recommendation.

**Reviewer**	**2**
**Name**	**Gopfert, Anya**
**Affiliation**	**University of Exeter**
**Date**	**09-Mar-2026**
**COI**	**None**

Thank you for sharing this study, which I have read with interest. I have a couple of main comments, that I hope may be addressable, but will require further work from the authorship team before publication.

- You are to be commended on a well written, and well presented study. I enjoyed reading it and definitely think it should be published, however I am not sure it is ready in current form for publication in BMJ open.

- I am concerned that all interviews are conducted within one hospital, and the serious limitation that this places on the validity of the results. In my opinion, in order to publish this study in BMJ open it would be important to have a wider range of participants to enable understanding of the wider perspectives, which may be used to influence behaviour and policy change more broadly. At present, with only staff interviewed in one hospital, it makes it hard to draw more generically applicable conclusions from the data generated. I am confident if further interviews were conducted, that this study would be much more publishable within an international journal. At present, I would argue that the findings are only applicable within the hospital where the interviews were conducted. I also do not think this limitation is adequately summarised within the text of the study - in terms of the impact that this limitation has on the study findings

- A minor point: I would propose amending 'top management' to 'senior management' for publication in English language journals as I think this phrasing would be more familiar to colleagues

---

VERSION 1 - AUTHOR RESPONSE

Reviewer: 1
Dr. Jennifer Runkle, North Carolina State University

Comments to the Author:
Hospital top management's understanding of and response to climate goals in healthcare: a qualitative study
Manuscript ID: bmjopen-2025-112882

**Reviewer comment**	**Response**
General Comments:<br>The thematic analysis and resulting themes effectively capture the practical challenges associated with setting and operationalizing climate goals within hospital systems, particularly those linked to financial incentives and reimbursement. The qualitative interviews provide a compelling picture of the real-world tensions between pursuing ambitious versus "low-hanging fruit" climate goals, the potential for unintended consequences, and the complexity of coordinating sustainability efforts across systems beyond the hospital itself. The manuscript also appropriately highlights the friction between climate action and competing organizational pressures such as economic constraints, staffing shortages, and immediate clinical priorities. Although the paper only focuses on one hospital system, the key advantage is that goals have been adequately set and tied to reimbursement, much further than most hospital systems and results reveal a sobering picture of the pitfalls of implementation. This paper will be of interests to BMJ's readership and help guide other health systems in the process of agreeing and acting upon climate goals.<br>Abstract:<br>The abstract is clear, well aligned with the study objectives, and accurately reflects the study design, key findings, and implications.<br>Introduction:<br>Fantastic introduction. The introduction is strong, well written, and comprehensive. It effectively situates the study within the global movement toward net-zero healthcare systems, clearly articulates the leadership role of healthcare managers in reducing greenhouse gas emissions, and convincingly establishes the literature gap regarding decision-makers' perceptions and implementation challenges. The rationale for the study is well justified.<br>Methods:<br>This paper cites an important case example of a Swedish Healthcare System where the national climate policy outlined by the Swedish Parliament, was translated and defined as regional long-term goals and specified into regional climate and sustainability	We are truly grateful for your generous appreciation of our work and for taking the time to engage with the manuscript so thoughtfully. It means a great deal to us that the strengths of the study were recognised, and we deeply appreciate your supportive and affirming reflections.

goals by the Regional Council and further translated into yearly pay-for-performance indicators set by the Regional Health and Medical Administration. These pay-for-performance goals were then turned into hospital-specific goals and approved by the hospital; if goals were not achieved by the end of the year then reimbursement would be half of the total contract value. Top managers at the selected hospital system were responsible for environmental progress reviews and were selected to be in the study is well justified. The inclusion of reflexivity was an added advantage towards the study design, enhancing transparency.	
Results:<br>Five themes emerged and are clearly described.<br>Figure 1: The figure is currently too small to read and would benefit from improved resolution and layout. In addition, the caption should be revised to better support the figure as a stand-alone element. Clarification of the intended audience (e.g., hospital leadership vs. the broader healthcare system) would also strengthen the figure's interpretability and impact	Thank you for noticing us about this. We have now revised the figure (please the new uploaded version) and the captions (please see p. 15).
Discussion:<br>The discussion is thoughtful and appropriately emphasizes the need to move beyond siloed thinking and uncertainty by implementing monitoring systems that link climate actions to emissions, population health, and costs. However, this recommendation reads as quite ambitious at present. The manuscript would be further strengthened by providing more concrete examples of what the input data for such monitoring systems might look like in practice and how these data could realistically be scaled to the hospital level. Additional consideration of feasibility, data infrastructure, and resource requirements would enhance the policy relevance of this recommendation.	Thank you for providing insights on how to strengthen the manuscript! Please see p. 17-18 on how we have developed this!

Reviewer: 2

Dr. Anya Gopfert, University of Exeter

Reveiwer comment	Response
Comments to the Author:<br>Thank you for sharing this study, which I have read with interest. I have a couple of main comments, that I hope may be addressable, but will require further work from the authorship team before publication.<br>- You are to be commended on a well written, and well presented	Thank you very much for your positive and encouraging comments on our work. We appreciate your thoughtful engagement with the manuscript and are grateful that you found value in this.

study. I enjoyed reading it and definitely think it should be published, however I am not sure it is ready in current form for publication in BMJ open.	
- I am concerned that all interviews are conducted within one hospital, and the serious limitation that this places on the validity of the results. In my opinion, in order to publish this study in BMJ open it would be important to have a wider range of participants to enable understanding of the wider perspectives, which may be used to influence behaviour and policy change more broadly. At present, with only staff interviewed in one hospital, it makes it hard to draw more generically applicable conclusions from the data generated. I am confident if further interviews were conducted, that this study would be much more publishable within an international journal. At present, I would argue that the findings are only applicable within the hospital where the interviews were conducted. I also do not think this limitation is adequately summarised within the text of the study - in terms of the impact that this limitation has on the study findings	Thank you for this thoughtful comment and for carefully engaging with our methodological choices. We appreciate the opportunity to clarify why the study includes interview data from only one hospital. Our decision was driven by the purpose to generate in-depth, contextually grounded insights. Focusing on a single hospital allowed us to explore how study participants understood and responded to climate gools within a coherent organizational setting. This depth would have been difficult to achieve with a multi-site design, which may risk a shallower engagement with each context. Including study participants from one hospital thus provided us with in-depth understanding, and we could identify variations and patterns within one organization that potentially influence the achievement of climate goals.<br><br>What is important to notice also is that our findings to not point to one shared understanding among the members of the senior management team. Multiple perspectives were identified and are synthesised into a conceptual model—the virtuous and vicious cycles—which may be used to guide the design and analysis of future multi-case studies. Moreover, as we are progressing with our research, the findings were presented to multiple stakeholders within the regional health system and internationally, who found resonance with the empirical findings from the specific hospital studied here.<br>We have revised the discussion section to thoroughly discuss the pros and cons of our methodological choice. We hope that the revised discussion will help the reader to reflect on the transferability of our findings.<br><br>Please see p. 18-19 for our suggested changes based on your comment, and we thank you so much for your valuable insights!
- A minor point: I would propose amending 'top management' to 'senior management' for publication in English language journals as I think this phrasing would be more familiar to colleagues.	Thank you for noticing this. We have changed according to your suggestion throughout the manuscript.

Reviewer: 1
If you have selected ‘Yes’ above, please provide details of any competing interests.: none to report

Reviewer: 2
If you have selected ‘Yes’ above, please provide details of any competing interests.: NA
